# Identifying transformational space for transdisciplinarity: using art to access the hidden third

**DOI:** 10.1007/s11625-018-0644-4

**Published:** 2018-11-13

**Authors:** Toddi A. Steelman, Evan Andrews, Sarah Baines, Lalita Bharadwaj, Emilie Rose Bjornson, Lori Bradford, Kendrick Cardinal, Gary Carriere, Jennifer Fresque-Baxter, Timothy D. Jardine, Ingrid MacColl, Stuart Macmillan, Jocelyn Marten, Carla Orosz, Maureen G. Reed, Iain Rose, Karon Shmon, Susan Shantz, Kiri Staples, Graham Strickert, Morgan Voyageur

**Affiliations:** 1 Nicholas School of the Environment, Duke University, 9 Circuity Drive, Durham, NC 27708-0328 USA; 2 School of Environment, Resources and Sustainability, University of Waterloo, Environment 2, 200 University Avenue West, Waterloo, ON N2L3G1 Canada; 3 School of Environment and Sustainability, University of Saskatchewan, Room 323, Kirk Hall, 117 Science Place, Saskatoon, SK S7N 5C8 Canada; 4 Deninu K’ue First Nation, Box 204, Fort Resolution, NT XOE OMO Canada; 5 Department of Environment and Natural Resources, Government of the Northwest Territories, 5102 50th Ave, P.O. Box 1320, Yellowknife, NT X1A 3S8 Canada; 6 School of Public Health, University of Saskatchewan, 104 Clinic Drive, Saskatoon, SK S7N 5E5 Canada; 7 School of Environment and Sustainability, University of Saskatchewan, 326 Kirk Hall, 117 Science Place, Saskatoon, SK 57N 5CB Canada; 8 Métis Local 125, Box 547, 105 Mcdonald Street, Fort Chipewyan, AB T0P1B0 Canada; 9 Traper and Fisherman, 525 Crate Ave, PO Box 69, Cumberland House, SK SOE-OSO Canada; 10 School of Environment and Sustainability, University of Saskatchewan, Toxicology Centre - Room 215, 44 Campus Drive, Saskatoon, SK S7N 5B3 Canada; 11 Charlebois Community School, Cumberland House, SK S0E 0S0 Canada; 12 Resource Conservation, Wood Buffalo National Park, 149 McDougal Rd, Fort Smith, NT X0E 0P0 Canada; 13 Mikisew Cree First Nation, Fort Chipewyan, AB TOP-1BO Canada; 14 The Department of Drama, University of Saskatchewan, John Michell Building - Room 189, 118 Science Place, Saskatoon, SK S7N 5E2 Canada; 15 School of Environment and Sustainability, University of Saskatchewan, 335 Kirk Hall, 117 Science Place, Saskatoon, SK S7N 5C8 Canada; 16 Department of Drama, University of Saskatchewan, John Mitchell Bldg., Room 141, 118 Science Pl., Saskatoon, SK S7N 5E2 Canada; 17 Publishing, Gabriel Dumont Institute of Native Studies and Applied Research, #2 - 604 22nd Street West, Saskatoon, SK S7M 5W1 Canada; 18 Department of Art and Art History, University of Saskatchewan, 3 Campus Dr., Saskatoon, SK S7N 5A4 Canada; 19 School of Environment, Resources and Sustainability, University of Waterloo, 200 University Avenue West, Waterloo, ON N2L 3G1 Canada; 20 Box 366, Fort Chipewyan, AB T0P 1B0 Canada

**Keywords:** Transdisciplinarity, Transformation, Art, Boundary objects, Hidden third, Respect

## Abstract

A challenge for transdisciplinary sustainability science is learning how to bridge diverse worldviews among collaborators in respectful ways. A temptation in transdisciplinary work is to focus on improving scientific practices rather than engage research partners in spaces that mutually respect how we learn from each other and set the stage for change. We used the concept of Nicolescu’s “Hidden Third” to identify and operationalize this transformative space, because it focused on bridging “objective” and “subjective” worldviews through art. Between 2014 and 2017, we explored the engagement of indigenous peoples from three inland delta regions in Canada and as a team of interdisciplinary scholars and students who worked together to better understand long-term social–ecological change in those regions. In working together, we identified five characteristics associated with respectful, transformative transdisciplinary space. These included (1) establishing an unfiltered safe place where (2) subjective and objective experiences and (3) different world views could come together through (4) interactive and (5) multiple sensory experiences. On the whole, we were more effective in achieving characteristics 2–5—bringing together the subjective and objective experiences, where different worldviews could come together—than in achieving characteristic 1—creating a truly unfiltered and safe space for expression. The novelty of this work is in how we sought to change our own engagement practices to advance sustainability rather than improving scientific techniques. Recommendations for sustainability scientists working in similar contexts are provided.

## Transformational spaces for transdisciplinarity

Transdisciplinarity is a response to perceptions that conventional, curiosity-driven science has been ineffective in dealing with contemporary sustainability challenges (Klein 2001). A transdisciplinary approach aims to combine scientific experience with other kinds of knowledges from collaborative partners to more effectively identify problems and solutions for pressing sustainability challenges (Scholz 2011; Lang et al. 2012; Scholz and Steiner 2015). However, co-producing knowledge can be difficult especially when dealing with diverse cultural values, knowledge systems, and ways of knowing (Kates et al. 2001; Cash et al. 2003; Crona and Parker 2012; Steelman et al. 2015). Technical scientific expertise is necessary to understand sustainability problems, but recognizing, acknowledging and respecting socio-political history, community-centered knowledge, expertise and wisdom and community values are equally important if researchers are to find transformative spaces for respectful exchange and progress (Nicolescu 2010; McGregor 2015).

In this article, we sought to identify transformative transdisciplinary spaces that created opportunity for respectful interaction for diverse parties to identify problems and work on solutions. We drew from Nicolescu’s theory of the “hidden third space”, which theoretically bridges the duality of a scientifically objective world view (Elias 1956; Berman 1981) and a subjectively experienced world view (Goffman 1959; Franks 2010). We use these words with caution as we recognize that knowledge from both western science and indigenous perspectives may be both objective and subjective. We used art as a boundary object, something that holds individual and common meanings to interacting people engaging with each other cooperatively (Trompette and Vinck 2009; Timmermans 2015). Art, which often relies on intuition in both processes of creation and responsive engagement, can connect subjective experience with knowledge gained through implicit learning, using evocative visual forms to bridge logic and emotion (Leiberman quoted in De Wolf and Lumer 2017:13). We operationalized these concepts through the example of the Delta Dialogue Network—a multi-year transdisciplinary research project involving indigenous peoples from three inland delta regions in Canada, territorial and federal government partners, and a team of interdisciplinary scholars, artists, and students.

## Transdisciplinarity, objectivity, and subjectivity

Transdisciplinarity has two dominant meanings (Nicolescu 2010). The first embraces the across and between notions of “trans” for how disciplines are approached—situating transdisciplinarity into a disciplinary framework to achieve a “superior stage of disciplinarily” (Nicolescu 2010: 20). The second takes “trans” to mean beyond and thus indicates moving beyond disciplines (Nicolescu 2010).

A further distinction exists within this second definition—one that turns on the difference between improving science versus transforming knowledge. Transdisciplinarity as expressed as part of mode-2, post-normal science (Funtowicz and Ravetz 1993; Nowotny et al. 2001, Gibbons et al. 1994), seeks to improve science and research by including external communities in the definition of research problems and solutions (Klein 2001; Lang et al. 2012; Scholz 2011; Scholz and Steiner 2015). While differentiated from conventional investigator-driven, mode 1, disciplinary science, this definition constrains the transdisciplinary experience to an exploration of extending scientific investigation only into a limited social sphere—involving participants who are outside of the academy. Nicolescu (2010) argues that this practice of transdicisiplinarity precludes consideration of other dimensions of subjective human social experience, such as intuition, relationships, and spirituality. Arguably, such an approach also excludes other forms of knowledge that may be grounded within these experiences or dimensions, and privileges western science as a dominant way of knowing.

Modern science has attempted to separate objective experiences from subjective experiences (Nicolescu 2010). It has also fragmented knowledge into disciplines. The separation of objectivity and subjectivity reduces what is knowable to primarily that which can be objectively verified, thereby depleting and invalidating other more subjective lived experiences and impoverishing our ability to fully express what is important in our lives. In part, what is missing is the potential to acknowledge and respect emotional resonance, relationships, and spirituality or what connects us as human beings to the rest of the world, and beyond, what some indigenous elders have referred to as when the mind and heart come together (MacColl). This connection is represented by what some have identified as the transrational (Barrett 2013). Transrational knowing is often experienced as sensation, vision, or dream (Barrett 2013) and has been expressed by indigenous peoples as a legitimate form of knowledge generation that can contribute to co-production of knowledge for sustainability science (Kealiikanakaoleohaililani and Giardina 2016).

Both the objective and subjective are important in how we make sense of our environment, lives and experiences, and they interact in what Nicolescu (2012: 21) identifies as the “hidden third”. Drawing inspiration from quantum physics to suggest that all matter exists in dual states of impermeability and permeability, Nicolescu (2010:34) proposes that this theoretical “hidden third” space creates opportunity for bringing together both the subjective and objective within a larger umbrella of meaning. In this way, it creates space for intuitive knowledge in that it dispenses with conceptual categories, reasoning, and the separation of subject and object (Iverson 2017). Creating space for intuition, as is increasingly recognized in the emerging fields of neuroscience and cognitive psychology, is described as a holistic process that synchronizes unconscious contents of the brain and brings these into consciousness (de Wolf and Lumer 2017). The “hidden third” is a border zone or transition, where creative energy gives shape to what is beyond words (Vandenbroeck 2017). It is a more holistic and representative of the entirety of human experience (Nicolescu 2010; Little Bear 2011).

While Nicolescu is long on theory and approach, he is short on examples. Nonetheless, his work raises important questions about the places, where we can find the hidden third, what the hidden third could look like, how we could practice transdisciplinarity if we could find the hidden third, and how wisdom gained in the hidden third could contribute to transformative change towards social–ecological flourishing. As his writing suggests, a key criterion for identifying this space would be one where the objective and subjective could come together.

## Art, transdisciplinarity, and boundary objects

Art in a transdisciplinary context differs from other uses of art in scientific research. For instance, there is a history of using art to “prettify” science, where artists serve a design function for scientists to aid in translational goals (Ede 2005: 3). There are also traditions of “eco-art” that celebrate the natural environment and outdoors (Adams and Chisholm 1999; Beardsley 2006; Berleant 2002; Grande 2004; Moyer and Harper 2011) and “socially engaged practice” that is more politically motivated to address social, environmental, or other sustainability issues (Bishop 2012; Brown 2014; Demos 2016; Finkelpearl 2013; Kester 2013; Thompson 2012; Zurba and Berkes 2014; Zurba and Friesen 2014; Lineberry and Wiek 2016).

Art has also enhanced teaching through deeper emotive and sensory experiences (Marshall 2014; Jacobson et al. 2016; Jacobson et al. 2007) including community and participatory theater (Strickert and Bradford 2015; Brown et al. 2017). Collaborative art has been used to understand diverse values against the backdrop of changing socio-cultural environments (Billings et al. 2007; Zurba and Berkes 2014; Zurba and Friesen 2014), and to understand socio-ecological change (Bradford and Bharadwaj 2015; Rathwell and Armitage 2016). Artistic expression can connect researchers with more subjective emotions as well as multiple senses in support of intuitive “perceptions of coherence” (De Wolf and Lumer 2017:16) that might respect different ways of knowing and enable connection to the hidden third space in transdisciplinary practice.

Art is one avenue for bridging different ways of knowing, since it holds potential for creating boundary objects with multiple meanings and interpretations for diverse audiences (Rathwell and Armitage 2016; Halpern 2012; Singh 2011). As boundary objects, artistic works can breathe life into Nicolescu’s hidden third. Boundary objects are “any artefact which is involved in coordination between actors or which is at the boundary of two worlds” (Trompette and Vinck 2009, p. 7). They are devices used to initiate, and create conversation and support decision making for diverse and sometimes divided groups or cultures with different knowledge systems such as scientists and practitioners, local people, and policy writers (Star and Griesemer 1989; Crona and Parker 2012, Van Pelt et al. 2015). The process involved in boundary object use includes three steps; generation of ideas, standardization attempts, and the actual creation of boundary objects (Leigh Star 2010). Although initially presented as cyclic in nature, the process of creating and using boundary objects is now understood as blurry (Scoles, 2018). The use of boundary objects creates an operating space between different ‘social worlds’ in which stakeholders can explain their interpretations without the need for consensus while adapting an object to meet a goal (Shackley and Wynne 1996; Star and Griesemer 1989; Leigh Star 2010). Boundary objects are used across disciplines and have included items such as data, like climate parameterizations (Sundberg 2007), functional system maps (Beckett, 2015), or participatory art (Zurba and Berkes, 2014).

Rathwell and Armitage (2016) catalogue the mechanisms through which art can function as a boundary object and these include: (1) embedding knowledge, practice, and belief into art objects; (2) sharing knowledge through the language of art; (3) sharing of art-making skills; (4) engaging art as a contributor to monitor social–ecological change; (5) employing art to foster continuity through time; and (6) placing art as a site of knowledge co-production.

By arising from, and provoking in viewers, intuitive connections, art can bring together unconscious and conscious knowledges in forms that embody memory, perception, emotion, and kinetics (De Wolf and Lumer 2017). Art holds the potential to use multiple literacies (Eisner 1991), cross cultures, and create intergenerational touchstones through key material objects and practices. Art is accessible and holds the potential for non-expert involvement. It also can move scientists away from their own scientific traditions in problem-framing, a common limitation to transdisciplinary practice (Brandt et al. 2013) and an identified benefit of boundary objects (Trompette and Vinck 2009). Material cultural objects as part of art can be important, because they represent key aspects of history and encapsulate a variety of meanings (Athayde et al. 2017). Auditory, visual, tactile, and kinetic experience can capitalize on multiple senses. Design that emphasizes social interaction, participation, and the opportunity to share can create relational experiences. Understanding the role of art leads to two additional criteria in the identification of transformative knowledge space—interactive experiences and opportunities for multiple sensory experiences.

## Respecting different knowledge traditions

Transdisciplinarity rests on the premise that different participants can come together to define problems and solutions for sustainability. In some instances, the operating world views of diverse participants may be closely aligned, especially if the basis of knowledge and knowing is culturally shared. Increasingly in contemporary society, the basis for knowledge and knowing seems to be fraying, with decreasing trust in institutions in general and science in particular (Gauchat 2012). Nerone (2017) puts the debate about contemporary societies’ dependence on unidirectional social media videos, and popular sources of information succinctly by stating the “… gap between the many interests of people and the fictional wisdom of the public is the characteristic problem of modern democracy” (p. 204), while Sunstein et al. (2016) discuss how increasingly rigorous and precise climate research polarizes public beliefs, rather than unites them towards taking action; a troubling trend. Therefore, insights into how we can find respectful space for exchange and interaction about sustainability problems are pressing (Wynne 2006). We explore knowledge divides in an extreme case with indigenous communities who have strong relationships with their environments and long histories of knowledge that has been passed down from generation to generation (Moeke-Pickering et al. 2006), but also have had long and fractured relationships with colonial institutions and representatives, including western science and scientists, respectively (Nadasdy 1999).

Indigenous communities, diverse in their own languages, knowledges, and outlooks, also may differ in their worldviews when compared with western scientists (Berkes et al. 1995; Houde 2007; Wilson 2008; Barrett 2013; Greenwood et al. 2017). A significant challenge in working in a transdisciplinary context includes how to appropriately represent and respect multiple disciplines, multiple ways of knowing, and diverse knowledges to overcome a sense of “otherness”. Bridging these divides requires opportunities for interaction, learning, and mutual trust building (Castleden et al. 2012; Caine et al. 2009; Tondu et al. 2014). Furthermore, it has been increasingly recognized at multiple levels, across communities, academies, governments, and others that indigenous knowledge and wisdom can and should play a key role in problem-solving, management and decision making together with other forms of knowledge (Agenda 21, as cited in Pohl et al. 2010; Berkes et al. 1995; Reid et al. 2006; Davis 2006; Raymond et al. 2010; Armitage et al. 2011; Tengö et al. 2014; Castleden et al. 2017). Thus, bridging knowledge systems in a meaningful, respectful, relevant, responsible, and reciprocal way is a critical policy issue, and efforts to further advance space for such bridging are needed (Kirkness and Barnhard 1991; Castleden et al. 2017).

Challenges to mutual understanding arise in part, because some of the characteristics of indigenous knowledge (traditional or modern) and western conceptions do not neatly align or map onto each other. Some differences that have been presented include the highly personal, experiential, and holistic nature of indigenous knowledge (Castellano 2002) and the more impersonal, compartmentalized nature of western science. In addition, the ownership of knowledge may be fluid, because often, its development arises from and is refined through social interactions (Bradford et al. 2016). In some indigenous communities, knowledge may not be written down, but communicated orally and only under special circumstances (Battiste 2002; Castellano 2002). Sources of indigenous knowledge can also include knowledge handed down over generations, empirical knowledge gained through observation and experience, and spiritually revealed knowledge acquired through dreams, visions, and intuitions (Castellano 2002). These characteristics do not easily lend themselves to presentation in conventional scientific expressions of knowledge—peer reviewed papers, or conference presentations. These scientific channels are themselves barriers to wider knowledge sharing (Giles and Castleden 2008; Bradford et al. 2016). Yet, western conceptions favouring analytical and/or reductionist methods, where objects of study are put in controllable experimental settings stand in stark contrast to indigenous knowledge systems through their poor ability to build solutions given their apparent separation from context and their adaptability to new contexts such as those arising from climate change (Nakashima and Roué 2002; Mazzocchi 2006; Cameron et al. 2015).

Transdisciplinary practice faces four dominant challenges to respecting the integrity of indigenous knowledge and its basis in tradition and experience. First, there is a tendency for indigenous knowledge and ways of knowing to be subjected to evaluation by western scientific standards (Nadasdy 1999; Brook and McLachlan 2005; Barrett 2013; Watson 2013). Western scientific knowledge has been the dominant paradigm for the last centennial for research and decision making (Davis 2006; Reid et al. 2006; Pohl et al. 2010) and indigenous knowledge has often had to conform to western standards or approaches in attempts to connect knowledge systems (Nadasdy 1999; Armitage et al. 2011; Berkes 2012; Tengö et al. 2014). Second, the identified overlap between western science and indigenous knowledge may be reduced to what is empirically observable, thereby neglecting and disrespecting a large portion of what is known by indigenous peoples (Battiste and Youngblood 2000; Simpson 2001; Houde 2007; Barrett 2013, see also Gagnon and Berteaux 2009 for examples). Third, there is a tendency to focus on the environmental and ecological aspects of indigenous knowledge, while other dimensions, including spiritual, relational, and emotional aspects, are ignored (Battiste and Youngblood 2000; Berkes 1999; Simpson 2001; Brosius 2006; Barrett 2013; McGregor 2004). Finally, indigenous knowledge is often mediated by people (researchers or others) outside of indigenous communities (Nadasdy 1999; Brosius 2006). An important element in addition to these challenges is the need to create a safe space, whereby people feel comfortable addressing cross-cultural misunderstandings and misperceptions. While Castleden et al. (2017) present 6 R’s (respect, relevance, reciprocity, responsibility, relationality, and reconciliation) as necessary components for research with indigenous people, we understood a safe space to mean a physical, emotional, and intellectual space that was “specific, local, and relevant” to participating indigenous nations (Greenwood et al. 2017, p. 186). That space also needed to embrace, rather than deny differing identities, and acknowledge that personal and collective cultures influence our perspectives and actions (Hall and Wilkes 2015). This is consistent with the teachings of Elder Willie Ermine of “ethical space”, whereby people of different cultures work together through dialogue that pays attention to cultural differences, hidden values, and intentions, and how these govern our behaviours (Ermine 2007: 202–203). Altogether, this suggests two criteria that may be relevant when identifying a dignified knowledge space for transdisciplinary transformation. Ideally, researchers and community members would create spaces for different world views that are safe and unfiltered.

## Hallmarks of respectful transformative transdisciplinary space

**Fig. 1:**
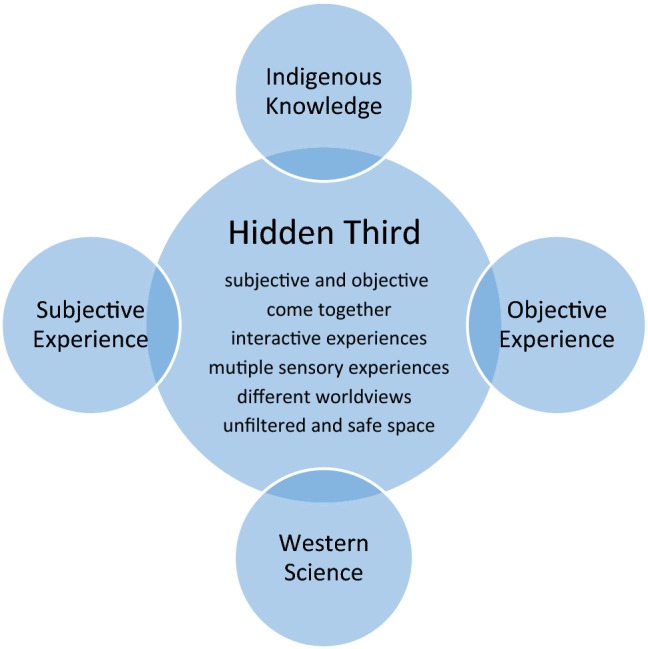
Conceptual transdisciplinary space

The literature review in the preceding sections suggests that five key characteristics typify a “hidden third” transformative transdisciplinary space, as illustrated in the central circle in Fig. 1. These characteristics include:

1. Evidence of the subjective and objective coming together (Nicolescu 2010, 2012; Little Bear 2011; Iverson 2017; De Wolf and Lumer 2017). This would include evidence of rational, empirical science as well as examples of spiritual, relational, and emotional expressions that have the power to make one feel a connection to what one is witnessing.
2. Interactive experiences that encourage individuals to share with others what they are experiencing—to breach the divide of “otherness” (Strickert and Bradford 2015; Brown et al. 2017).
3. Multiple sensory experiences that include sound, smell, sight, touch, and taste (Law 2004; Szerszynski 2003). Presentation would move beyond the rational, thinking brain to engage all the senses to gain enriched understandings by exploiting connectivity naturally present in the brain.
4. Space where people with different worldviews can come together without being validated by the other (Barrett 2013; Battiste and Youngblood 2000; Houde 2007; Berkes et al. 1995). For this article, this space would include the researchers and community participants, youth and elders, and scientists and non-scientists.
5. Unfiltered and safe space, where people can express their views and visions directly and not have them mediated by others (Nadasdy 1999; Brosius 2006; Castellano 2002). This would mean accommodating the knowledge system within which the knowledge is created to allow it to be expressed without fragmentation. People are comfortable expressing what they do not know and can have misunderstandings and misperceptions clarified.

## Context for research: delta days and the building bridges project

**Fig. 2:**
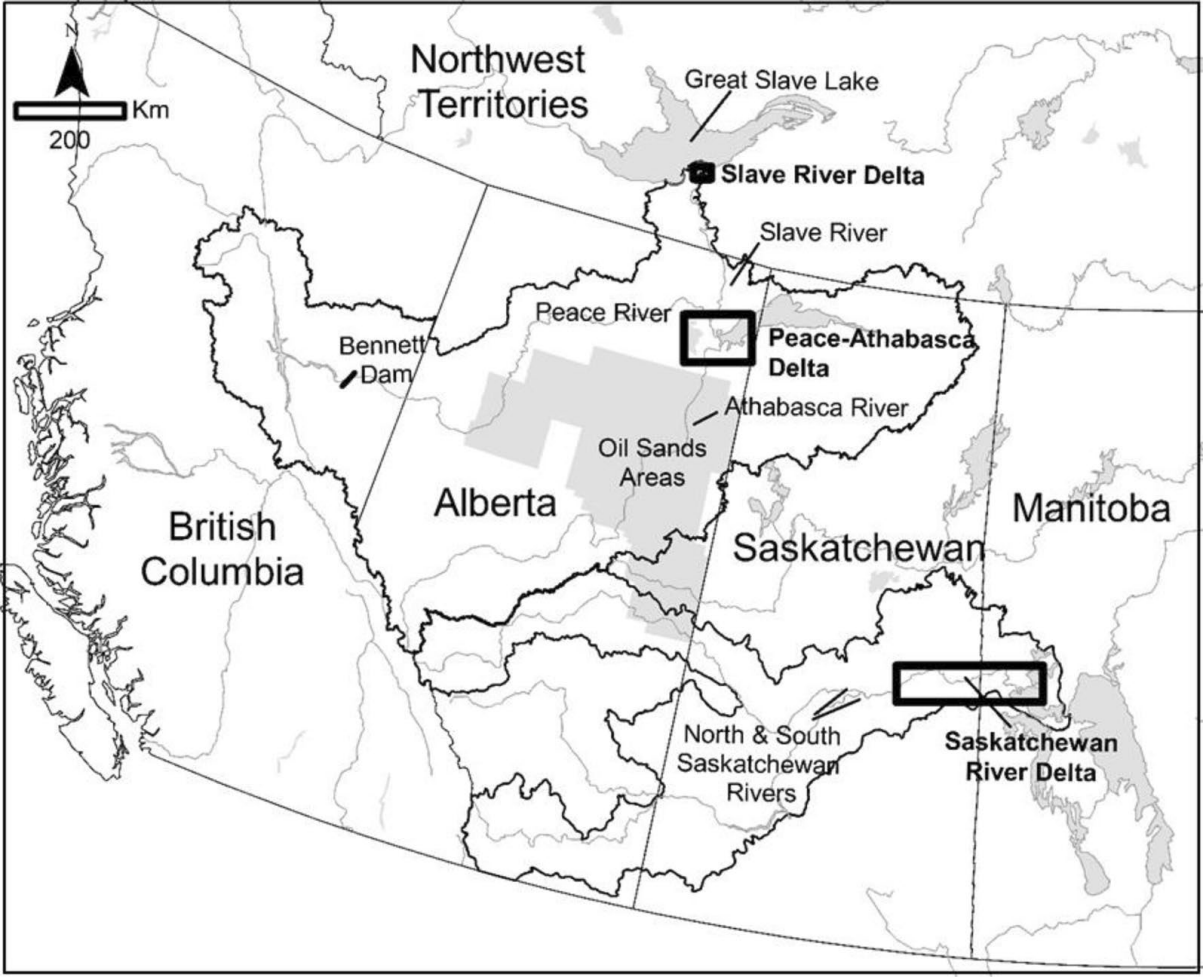
Three Canadian Inland Deltas. Created by Chrystal Mantika-Pringle

The delta dialogue network (DDN) is a transdisciplinary sustainability science project focused on co-creating knowledge with inland delta communities in northern Canada undergoing ecological and social change (see Timoney 2013; Schindler and Donahue 2006, Mantyka-Pringle et al. 2017). The DDN initiated collaborative processes with five communities in three inland deltas (see Fig. 2)—Cumberland House, Saskatchewan and Opaskwayak, Manitoba (Saskatchewan River Delta); Fort Chipewyan, Alberta (Peace-Athabasca Delta); and Fort Resolution, Northwest Territories and Fort Smith, Northwest Territories (Slave River and Delta) to synthesize, bridge, translate, and mobilize knowledge from existing research and community monitoring programs. The DDN was initiated in 2014 and sought to complement the significant work by community organizations, collaborative multi-party monitoring partnerships (such as the Slave River and Delta Partnership and Peace-Athabasca Delta Ecological Monitoring Program), and other researchers that had roots back to 2011. Knowledge generated by these efforts and others have included insights from community-based water monitoring programs (AANDC/GNWT 2012; Pembina Institute 2016), tracking contaminants in fish (Green et al. 2016; Ohiozebau et al. 2016), providing insights from indigenous knowledge (Wolfe et al. 2007; White 2006; Wesche 2009; Bradford and Bharadwaj 2015), measuring long-term hydrological change (Sagin et al. 2015), understanding the role of water in shaping place identity (Fresque-Baxter 2015), and power dynamics in decision making (Andrews et al. 2018).

From 2010 to 2017, various researchers invested time within individual communities to build positive research relationships by being present, communicating, respecting, building trust, making genuine collaborative efforts, and exchanging knowledge (Caine et al. 2009; Tondu et al. 2014). In 2016, we sought to create a respectful new space for interaction in what we called delta days, a gathering which allowed representatives from all the communities to come together, meet and deliberate about how their deltas were changing, the causes of those changes, the consequences for them and what could be done. During this ‘generational’ space, work was completed on developing and designing a traveling, interactive art exhibit, building bridges, to communicate key findings, insights, ideas, and emotions with communities who sent representatives to co-lead delta days. This work started with the western scientists and was reshaped by our indigenous partners as they reacted to it. The exhibit itself became a boundary object and collaborative product emerging from the transdisciplinary spaces created in building bridges and delta days.

## Results #1: evidence of a transformative transdisciplinary space and boundary object generation

### Delta days 2016

A 3-day event titled, “Building Bridges between Deltas: Crossing Knowledge and Cultural Divides”, took place from April 5th to 7th, 2016 in Saskatoon, Saskatchewan and included a 2-day networking workshop coupled with a public engagement and research symposium that culminated in the traveling, interactive art exhibit.^1^ In total, more than 100 participants from 15 Métis and First Nations’ organizations, three community organizations, several universities, one industry organization, three government agencies (provincial, territorial, and federal), and one environmental non-governmental organization attended. The group was made up of youth, elders, land users, community leaders, students, researchers, decision makers, and other rights holders and stakeholders involved with deltas and delta communities. Community members insisted on having elders, land users and youth present, as elders are the knowledge keepers, land users work and know the land, and the youth are the next generation to be educated and who will be responsible for the future stewardship of the deltas (see Andrews et al. 2018). Five delta communities across the Peace-Athabasca, Slave River, and Saskatchewan River deltas were represented. Planning for the meeting was a collaborative effort between community government partners, territorial and federal government partners and academic researchers and artists.

There were two main goals for delta days and building bridges. First, researchers and community members wanted to build connections among people living and working in the three deltas, something heard from community partners that it is an imperative to exchange and build knowledge. Through the emergent relationality (Castleden et al. 2017), community and research participants in DDN hoped community members could share experiences, concerns and lessons learned related to the changes their deltas were facing, such as community-identified impacts from climate change, upstream industrial development, and water flow regulation. Second, community and research DDN participants wanted community members to identify main concerns for their communities and deltas. The creation of a collective voice about these concerns could direct the attention of policy and decision makers towards the future of these regions. Our indigenous partners felt building bridges could be used to raise policy makers’ understanding of the impacts on the deltas. Policy briefs emerging from the exhibit, and interactions with senior government officials from the Government of the Northwest Territories and Parks Canada assisted with knowledge mobilization for policy making. This represented locally developed solutions as opposed to more top–down, duty to consult approaches.

**Fig. 3:**
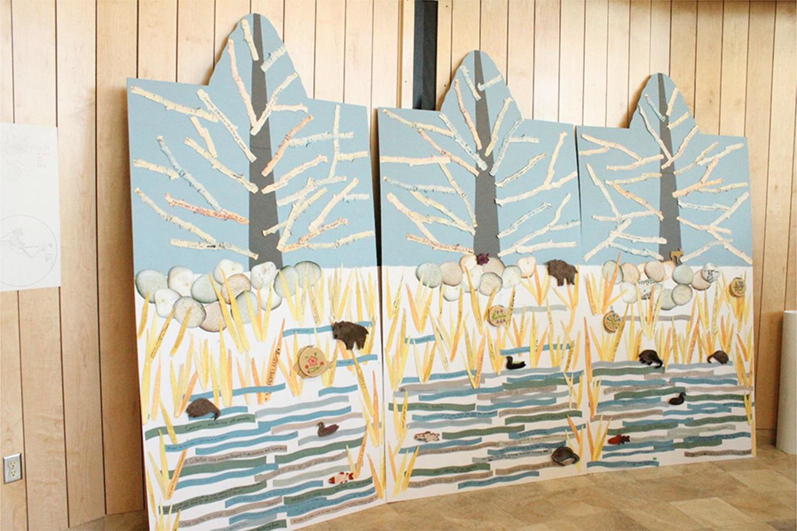
Delta days mural

**Fig. 4:**
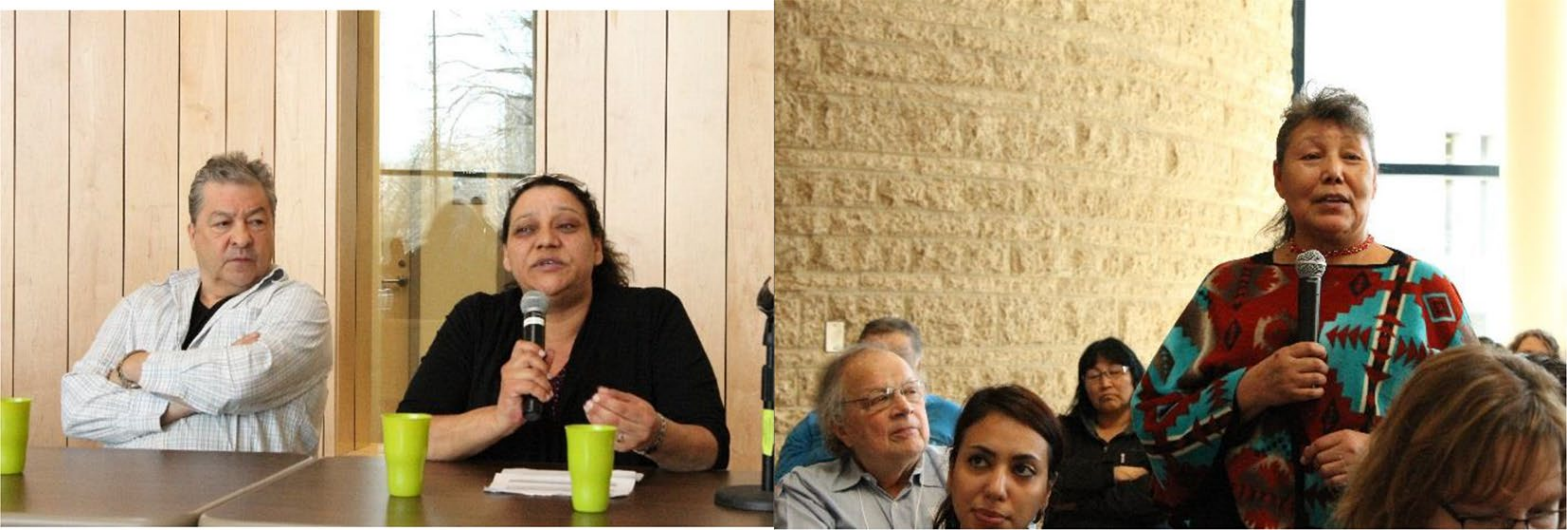
Community presentations

**Fig. 5:**
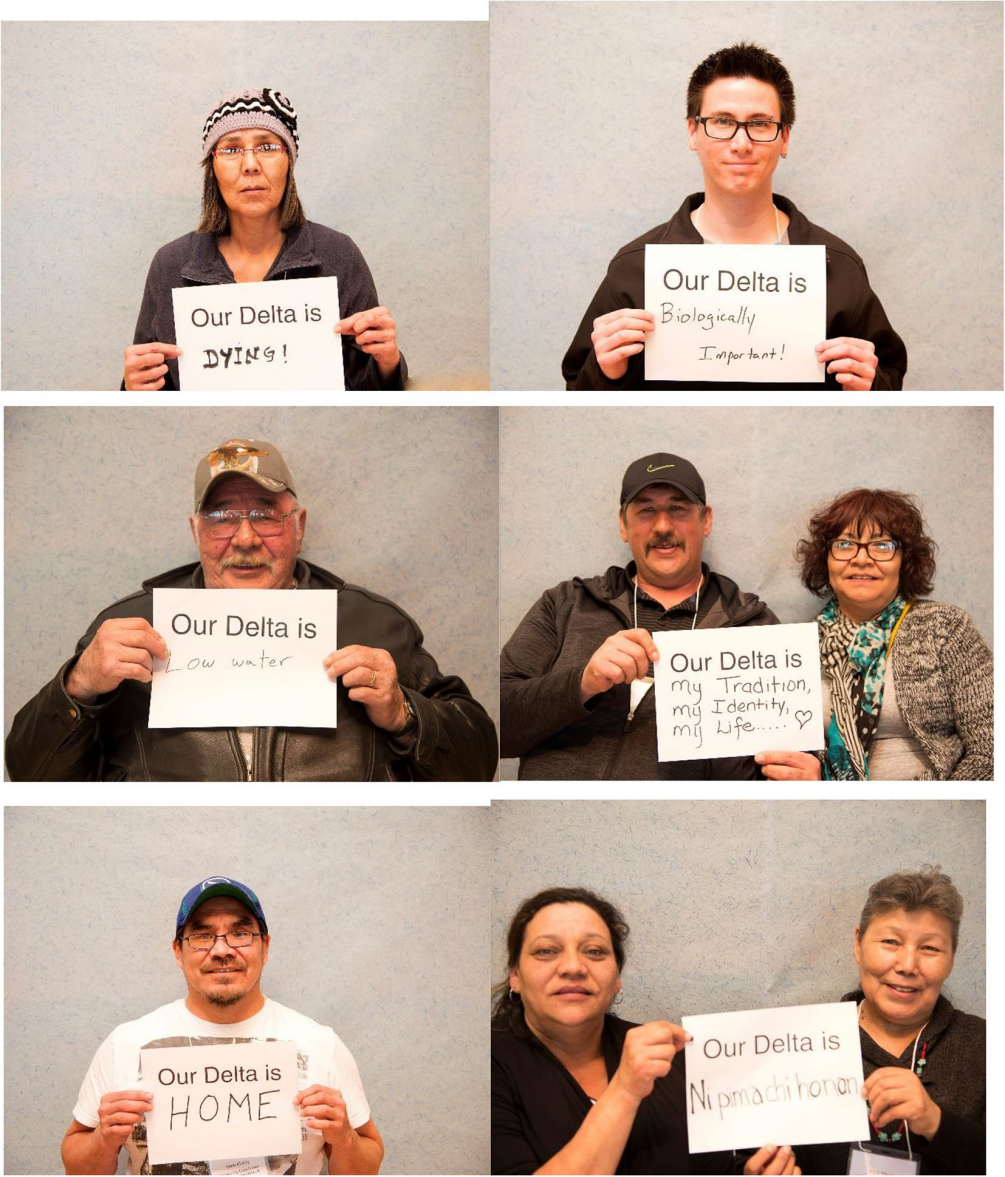
Photo booth examples

The delta days gathering included ceremonies, presentations, large and small group discussions, testimonials from elders and interactive group activities. Using visual materials and symbolic objects created in advance by the participating artist–researcher, based on earlier input from the collaborative team, the group activities built shared understanding as well as capacity for a subsequent traveling interactive art exhibit. Ceremonies, including a formal welcoming to territory, opening prayers, burning of sweetgrass, an honor song and elder responses led by indigenous leaders and elders representing the territories on which delta days occurred and the participating indigenous nations, were important to show respect and create a safe space for coming together of diverse worldviews. A key artefact from delta days was the creation of an initial temporary mural of delta life that gathered text and symbols generated during the workshops (Fig. 3). Throughout the event, we asked participants different questions about their deltas. The participants wrote down or drew their answers on artist-prepared “nature post-it” notes. The post-it notes were scans of marsh reeds, branches, stones and water, and were made to reflect the delta. Over the 3-day event, a group “delta portrait” developed as water, land and sky, was layered with the voices of the delta residents and researchers, and was gradually filled in. The mural was one means, where people could directly express their thoughts, feelings and reactions and locate them in unmediated space. Presentations were focused on three main topics which were determined by a committee of community representatives and university researchers. These included watershed planning, community-based monitoring, and youth engagement and were more objective, although the presentations also featured testimonials about loss, feelings, and sadness at the changes in ways of life and lifestyle in the deltas. These topics were chosen, because they represented existing efforts taking place in all three deltas that community members wanted to share with others and learn more about. The topics were laid out in an agenda to allow enough time for all issues to be covered, but constrained opportunities for more organic and emergent discussion. Representatives from each delta were given an opportunity to present on selected topics, focusing on what lessons or experiences they wanted to share with the other delta communities (see Fig. 4). Presentations were followed by reflections from elders, and questions and comments from the audience. A photo booth opportunity, led by a student artist, allowed participants to have their pictures taken and create a testimonial about their delta (Fig. 5). The portraits became digital slide shows at the center of the traveling exhibit, reminding residents at each delta of the connections they share and support they can lend each other around common issues.

**Fig. 6:**
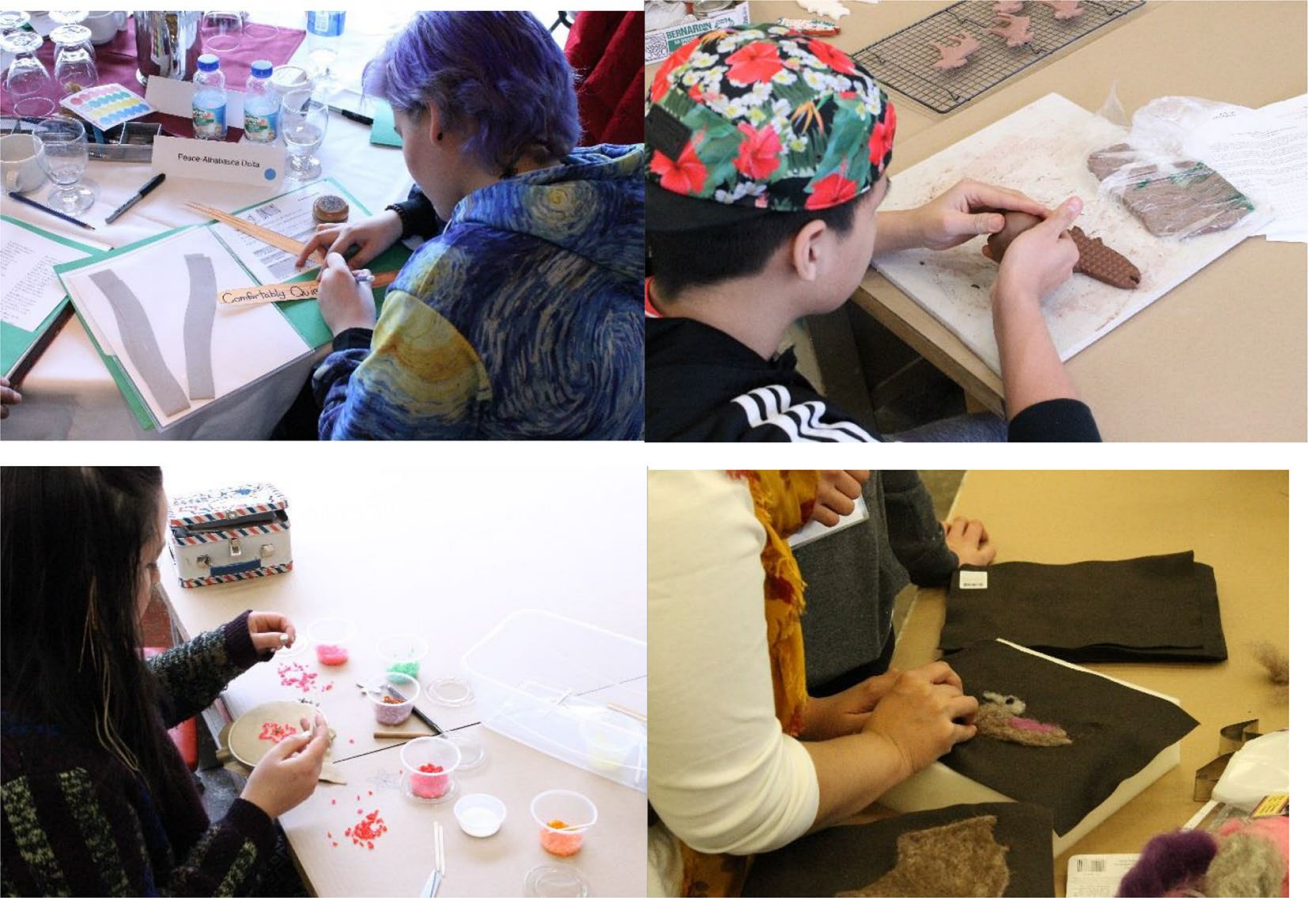
Youth art

To facilitate an opportunity with youth (ages 15–21) to explore and share their perspectives on the delta landscapes and their communities, youth participants chose to take part in both the main group activities and/or separate youth-focused activities (Fig. 6). The youth-focused activities were selected with advice from teachers and youth mentors in the communities and were designed to appeal to diverse interests (such as traditional games, fish-scale art, and finger weaving). Indigenous leaders recognized as being both educators and artists by their respective Nations led all aspects of the youth activities including preparation (e.g., respectful collection of fish scales, approval of teaching space), prayer, and the teachings as part of establishing a safe space without cultural appropriation. Career mentorship opportunities emerged when some of the youth engaged the artists in discussions about their experiences creating art-based careers as indigenous peoples.

## Results #2: birth of a traveling exhibit: standardization and boundary object creation

### Building bridges tour 2017

At the end of this 3-day delta day event, participants requested that the conversations taking place between different groups involved in the deltas continue. Community partners sent a clear message; they asked DDN community members and researchers to bring the knowledge from the Delta Dialogue Network back to their communities in a way that would get people talking about the deltas. In contrast to a conventional report, the purpose of the traveling exhibit was to use the artwork created from delta days to promote conversation and invite collaboration from wider community members. The intention was to celebrate the unique features of each delta while also gathering more stories, and telling the emergent broader account of how delta livelihoods in Canada were being affected, and what might be done to create greater community well-being in the face of change. This solidified message served as the standard from which the components of the exhibit were built. In this way, the boundary object’s standardization was embedded in community insights and narratives, rather than western empiricism.

**Fig. 7:**
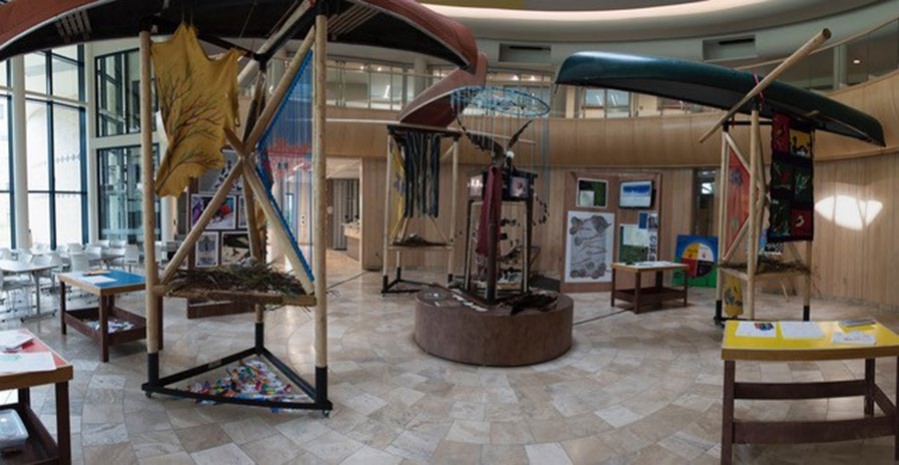
Building bridges traveling exhibit

**Fig. 8:**
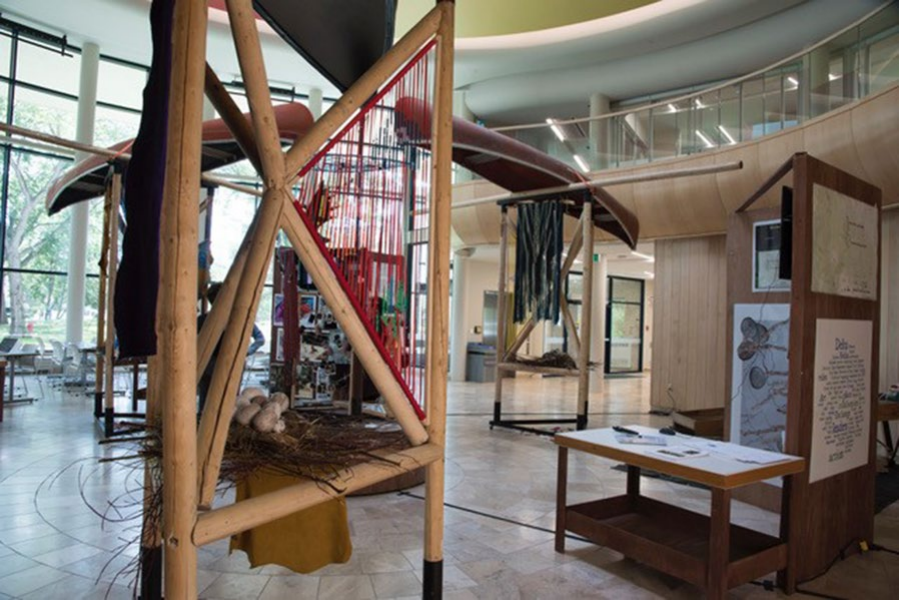
Examples of artefacts added as the exhibit traveled

The traveling exhibit took a mixed-media traveling installation format that combined different languages, indigenous knowledge, photography, recipes, word clouds, storytelling, and research findings (Fig. 7). The artist–researcher, along with a set design team from the Drama department and faculty consultants from SENS, incorporated community input from the delta days event and temporary mural to prepare the more robust and visually complex traveling exhibit. It was designed with advice from community representatives and structured so objects and images important to the deltas could be added to it as it moved to the various villages and towns (Fig. 8). This also allowed for re-engaging with the generation step as it traveled (Star 2010). In addition to connecting with the larger delta communities, this display sought to bring key messages to decision makers involved in the deltas. One of the main messages that was heard during delta days, and was expressed in the exhibit literally as text inscribed into the wood of each vertical structure and symbolically in the various media and objects included in the display, is: “Bring back nature’s flow, restore our deltas’ rhythms”. Two policy memos—one targeted for decision makers and one for a more general audience—were crafted to help communicate the policy advice from the various communities and represented standardization processes. These memos were handed out as the exhibit toured. The artistic content of the building bridges exhibit took many forms including visual, auditory, tactile and kinetic experiences.

Sculptures, videos, maps, photography, word clouds and posters were used to visually engage those who came to see the exhibit. Building bridges was made of three large periactoid structures made out of iron and wood frames—each of which were topped by an upended canoe. Artistic and cultural artefacts poured out of the canoes including: beadworks, animal skins, furs and nests with decoupage eggs created from the nature post-its from delta days. The center featured a large horizontal dream catcher from which hung a vertical helix of ribbon and Mallard feathers that was commissioned from Métis artist, Michela Carriere, a resident of one of the deltas. Grounded in the center was a smaller periactoid structure that had three screens featuring the photo booth portraits from delta days with messages “My Delta Is…” (Fig. 5). Videos depicted the delta day 2016 activities and were translated into English, Cree, and Chipewyan. A “Delta Ways Remembered” video (Bradford and Bharadwaj 2015) used whiteboard animation to tell stories from elders and local people from the South Slave region of the NWT.^2^ Maps illustrated the three inland river deltas.

As the exhibit toured, people continued to generate new ideas by bringing in photographs depicting delta life and aspects of that life that were important to them. Climate change information from research scientists was included that illustrated the climate across western and northern Canada has been warming and showing other clear changes in recent decades (Debeer et al. 2016). Charts were uploaded that displayed how rising temperature and the loss of cold continued to lead to declining snow and ice cover, changes in timing of freeze up and breakup of rivers, thawing of frozen ground, and changes to terrestrial ecosystems and altered water cycling (Debeer et al. 2016).

**Fig. 9:**
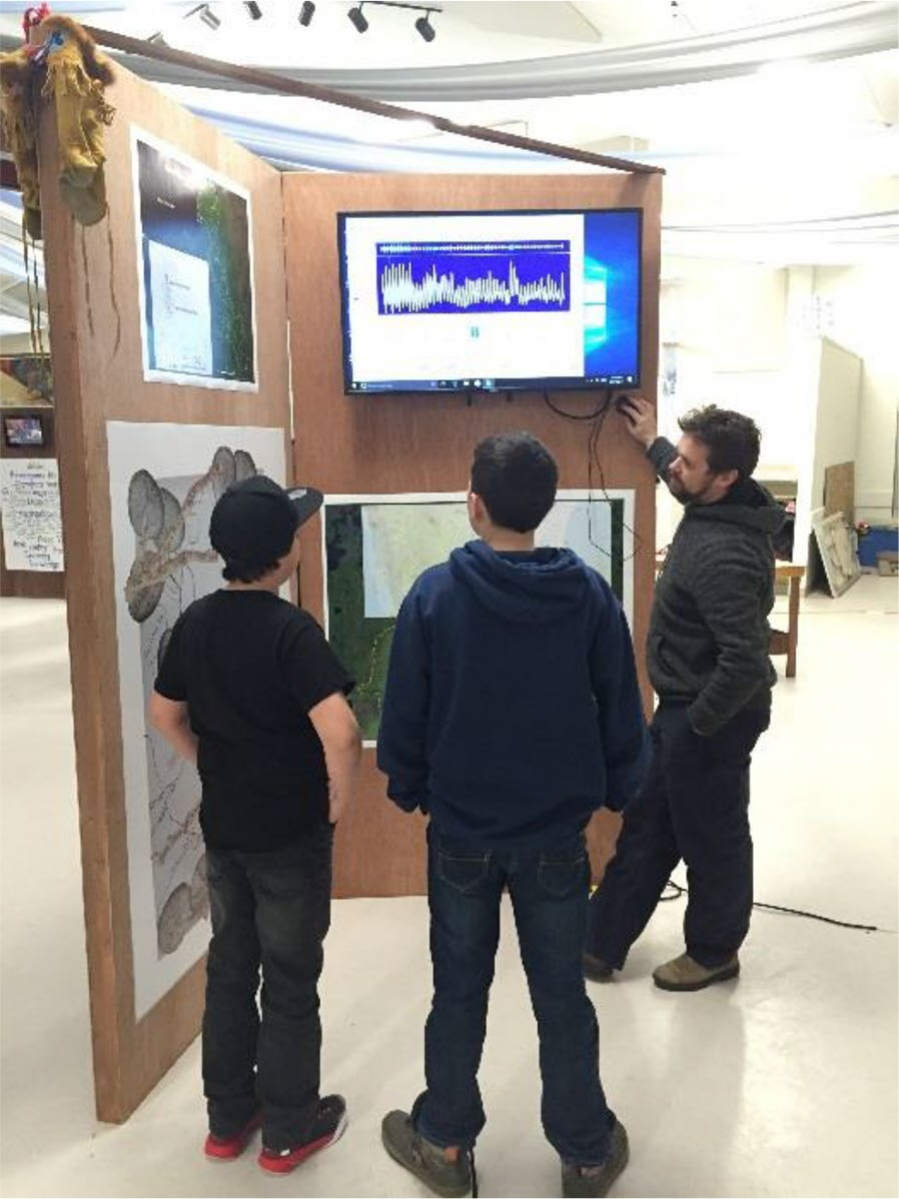
River heartbeat

Using sound, a listening station created a “heartbeat” of the three rivers that fed the deltas. Hydrographs from gauging stations that illustrate the amount of water flowing past the three deltas were translated into an audio file using Photosounder™. The listening station’s recordings highlighted changes before and after several dams were put in upstream in the 1960s, with regular annual pulses before the dams and weakened and erratic pulses after the dams. Headphones were used to listen to the rivers and were accompanied by an electrocardiogram-like visual display of the “heartbeat” of the river (Fig. 9).

**Fig. 10:**
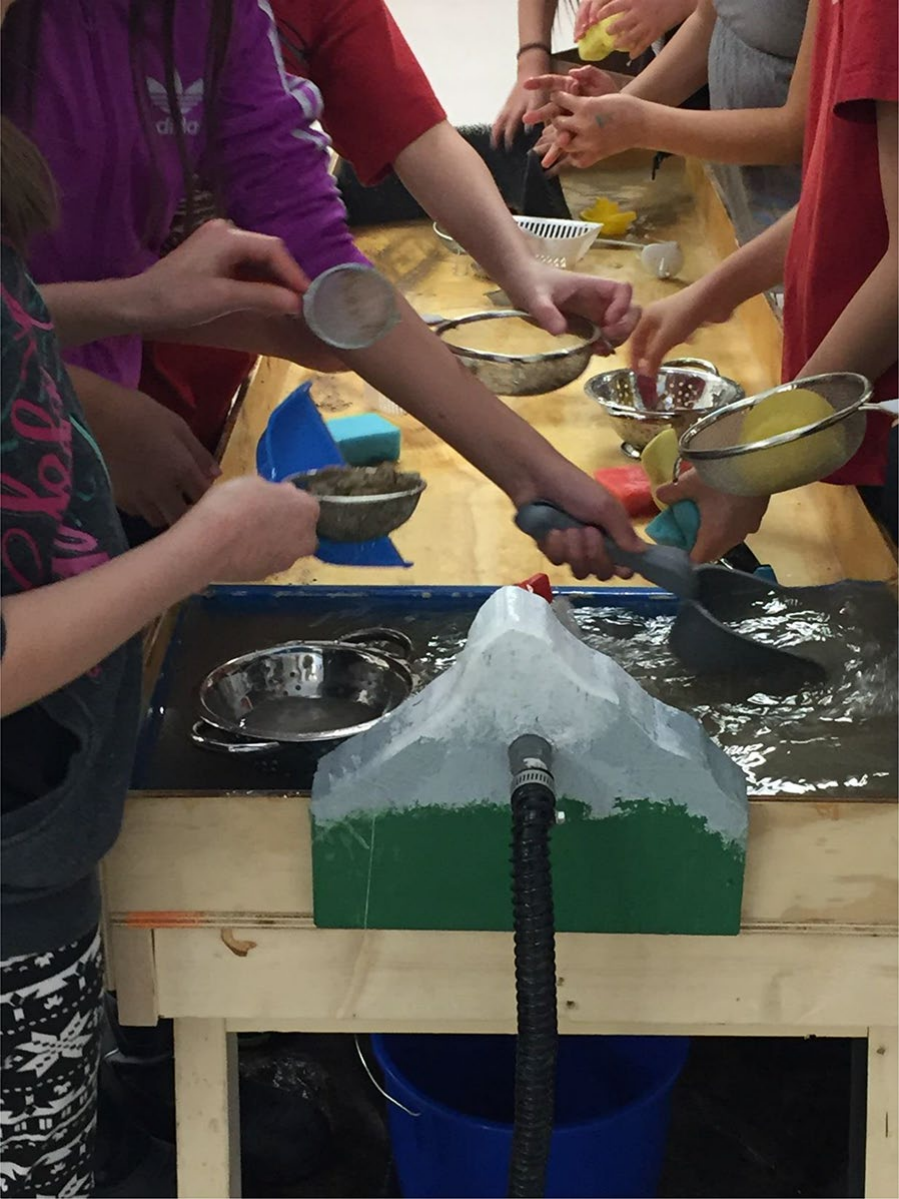
River in a box

Young people were also important generators of delta information through three additional components of the exhibit. A photo booth, coloring station, and a “River in a Box” were used as tactile experiences to involve people more actively in the exhibit. In the photo booth, people could opt to have their picture taken with a Polaroid camera while holding up a placard that testified what their delta meant to them. The coloring station was for children of all ages who could create art and have it displayed as part of the exhibit. The River in a Box was most dynamic (and messy) part of the exhibit, involving an interactive wooden table with a pump that mimicked a human-influenced river system (Fig. 10). It recirculated water flows into a reservoir, over a dam and into a sandbox. Children moved the sand, played with sieves and sand toys to experience how rivers behave and how deltas form as water slows down and deposit sediment. These activities created deeper understanding of what was happening to the deltas in some communities, including instigating a broader community discussion about the role of sediment in the Saskatchewan River Delta.

As people visited the exhibit, they brought artefacts that represented their own experiences in the delta or from their history. These included beaded moccasins, clothing, furs, trapping materials, and antique guns. People contributed recipes for preparing traditional foods from the deltas.

Various pieces of art were contributed. Decoupage eggs allowed us to reuse the nature post-its from delta days, by wrapping them around eggs that filled nests within the sculptures. A legacy art piece was created out of contributed art pieces to represent all three of the river deltas. This included the fish-scale art, felting, clay work, and finger weaving created during delta days by the youth who attended. This legacy art piece was reproduced as a framed print and sent to over twenty participants. It served to synthesize key messages and connect disparate groups and distant locations long after delta days and the building bridges tour ended and was indicative of a longer lasting and connective/symbolic legacy using art to connect multiple stakeholders.

**Fig. 11:**
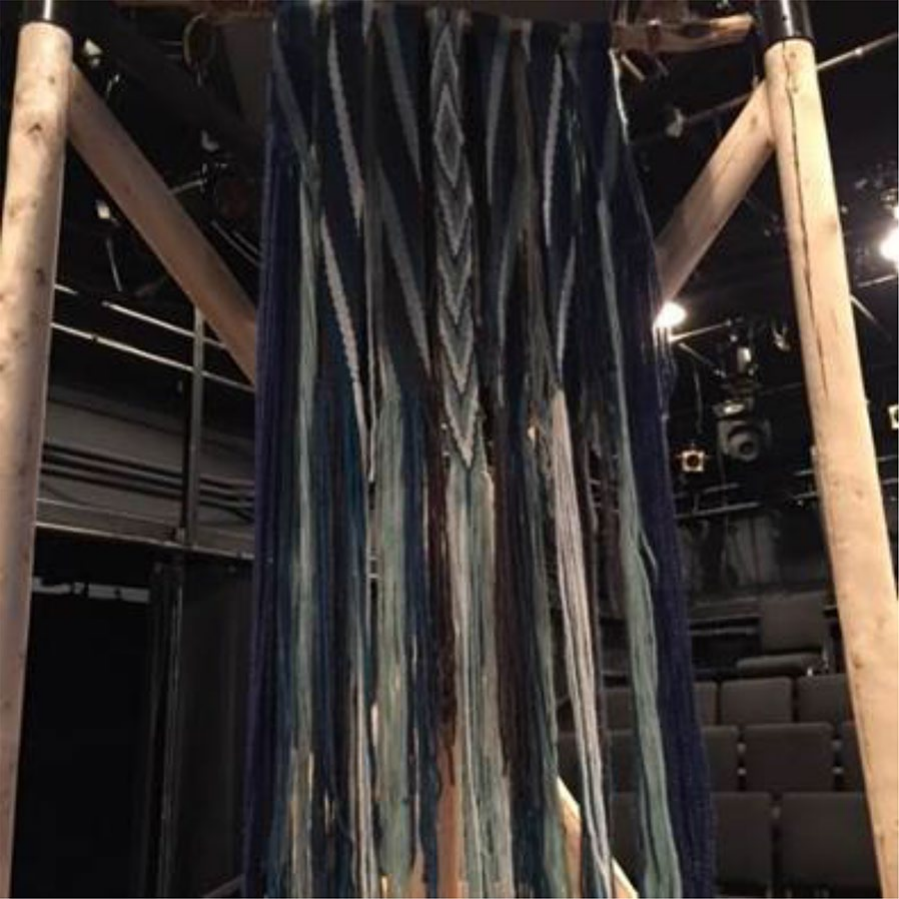
River song

The art was highly symbolic of the values important to those from the deltas. Components of the original mural created during delta days accompanied the exhibit. The Gabriel Dumont Institute, a Métis^3^ cultural institute, created and donated a finger weaving that was hand crafted by David Morin, Karon Shmon and David Werner, titled River Song (Fig. 11). The blue and grey weavings represented the waterways and were attached to a diamond willow walking stick. Paintings, felt blankets and sculptures were also contributed depicting the value of the delta for individuals and their communities.

The building bridges exhibit was well-received and well-attended. On the initial tour over 1000 people viewed the display in five different communities. This constituted 10–20% of the total community population in some places. After the tour, we were invited to host the display at the Western Development Museum in Saskatoon for 6 weeks (August 1st–September 15th, 2016). During that time more than 4000 people visited the museum. When people walked into the room for the first time and saw the display, by far the most common reaction was to request information about its origins from museum personnel, as well as touch, read, listen to, and watch what they could. Many people returned to visit with friends and family. People also asked for printed materials about the display. The attention grabbing display and the interactive components helped to convey the core message—bring back nature’s flows to restore our deltas’ rhythms. This message, conveyed from local communities to a broader public, signified that indigenous people still survive and are playing a key role in in creating their own solutions to move forward and gaining greater independence.

Because communities cared about having an impact on policy and decision makers, an effort was made to engage with these groups as well. Métis Councils, First Nation Chief and Councils, Mayors and Municipal Councillors and in the Northwest Territories the Premier and Members of the Legislative Assembly visited the display, to discuss what it represented and what might be done in response.

Another request from community partners was that nothing from the display be thrown away. The sculptures, furs, crafts and other donated pieces went back to the communities, the printed materials were archived at the University of Saskatchewan. The electronic equipment was used in a mobile decision laboratory. Two communities requested that parts of the display permanently reside in their communities and these requests were honored.

## Discussion: lessons for transdisciplinary practice

In this section, we detail the degree to which we met the hallmarks or criteria for developing a respectful, transformative transdisciplinary space. We illustrate how we were more successful with some than other criteria, as indicated by the plus and minus symbols in Table 1.

**Table 1:** Evidence of a transdisciplinary transformative space

	Delta days 2016	Building bridges tour 2017
Subjective and objective coming together	+ Ceremonies, large and small group discussion, researcher and community presentations on watershed planning, community-based monitoring and youth engagement, testimonials from elders, land users and youth.	+ Expressions of delta life in contributed artefacts (guns, furs, photographs, clothing, recipes for traditional foods), artistic works contributed by communities were symbolic of the value of the deltas, video of how things used to be in the deltas based on traditional knowledge/stories, scientific findings related to climate change, policy messages for decision makers
Interactive experiences	+ Large and small group discussion, artistic experiences	− While individuals could interact with aspects of the exhibit, it was primarily an individual experience. Groups and individuals could engage with each other as they walked through and experienced the exhibit
Multiple sensory experiences	− Art used to express concerns and opportunities for the deltas, as illustrated in the delta days mural. Youth engaged in multiple artistic activities—fish-scale art, finger weaving, and traditional games. A photo booth provided opportunity for visual testimonial	+ Listening station, River in a Box, videos, coloring stations, and photo booth were activities in which attendees to could participate
Different worldviews coming together	+ Opportunities for elders, youth, land users, researchers, students, government officials, non-profit organization representatives to present, discuss and comment	+ Different worldviews were fused together within the entirety of the traveling art exhibit
Unfiltered and safe space	− Direct opportunities for community members and researchers to express their views. An agenda constrained some of these opportunities due to time limitations. The agenda also forced topics for discussion instead of leaving it open-ended. This likely also created some fragmentation in how and why certain kinds of knowledge were presented	− University researchers designed the exhibit in consultation with community members. Content was directed by researchers. Designing space for organically adding to the exhibit as it traveled created opportunities for direct expression. Videos created opportunities for community members and elders to express themselves in their own words. All expression was in English and translated into other languages

As a boundary object, the display crossed disciplines, cultures and generations conveying a variety of meanings simultaneously to its varied audiences (Athayde et al. 2017). The design process, while constrained by distance from each of the three delta communities, utilized the initial 3-day workshops with key stakeholders to generate visual and textual input; these individuals and groups also had opportunity, by email and teleconferencing, to provide feedback on the traveling display as it developed under the guidance of the artist–design team. In this way, the process of boundary object creation was not limited to static phases, but instead sustained throughout the initial designing, and the tour.

On the whole, we were more effective in bringing together characteristics 2–5—the subjective and objective and allowing different worldviews to come together through delta days and building bridges—than in creating a truly unfiltered and safe space for expression- characteristic 1. Delta days was more effective for creating interactive experiences than building bridges. Building bridges were more effective at creating multiple experiences than delta days.

Items generated from both indigenous and western science worldviews were included in both spaces. Ceremonies, song, prayer, and testimonials were part of delta days. Material artefacts, art, testimonials, photographs and recipes that were part of delta life were included in building bridges. These expressions of the subjective helped contribute to a more emotional and spiritual experience during the events. For instance, comments written on the “nature post—its” and on signs held for the photo booth portraits captured participants heart-felt love of their delta as well as their frustration with ecological damage and loss of traditional life ways; these were part of both delta days and the building bridges exhibit. The sharing reminded participants of their driving virtues; honesty, environmental justice, and compassion were expressed as reasons for participating. Each attendee could experience the different knowledge types and make sense of them on their own terms as they viewed the exhibit. More objective science based findings were also presented in each event.

The variety of visual media mentioned here, generated by the artist–design team as well as community members, points to the range of multi-sensory and interactive experiences afforded by the traveling display. The “artist” in this case was not a singular individual but a responsive team who offered sketches, models and potential symbols, testing these with community members and science collaborators. For each of the artists, trained in more conventional, individualistic modes of artistic expression, the collaboration required a commitment to social engagement and belief in the role that art can play in contributing to environmental and political goals (Finkelpearl 2013; Kester 2013; Thompson 2012). The display included photos, maps, videos, wall texts and objects to be used, perhaps more akin to museum conventions than gallery ones (Bishop 2012), all incorporated beneath the dynamic space created by the three inverted canoes spilling their cargo of community-contributed objects and artefacts. As a complex object designed in response to requests from community members for something that would generate conversation and contribute to change, it did more than “prettify” science. Instead, it deliberately combined subjective and objective experiences, indigenous and scientific knowledges to breathe new life into the hidden third.

Different worldviews were also expressed in each event without being validated by the other. During delta days, opportunity for elders, youth and landowners/users to speak about their traditional knowledge and experience was given space, as was time for researchers both from the community and the universities to present their findings. Explicit discussions were held about the need to let both kinds of knowledge co-exist without the need to integrate them. These different worldviews were fused together into building bridges as part of the whole exhibit.

Both delta days and building bridges were less successful in creating truly unfiltered space for expression (Brosius 2006; Castellano 2002). While there were some opportunities for individuals to express their views and visions directly, much of the space was defined by researchers with the input from community members. Artistic expression was limited by time and facility constraints. Designed prototypes were modified by participants, taking the place of more open-ended creative processes. Language was an additional barrier as all communication was in English. In some instances, videos were translated into Chipewyan and Cree, but English remained the dominant language. Accommodating indigenous knowledge systems without fully engaging in the languages in which these knowledge systems exist was and will likely continue to be a significant barrier in creating unfiltered and safe space for expression and interaction.

Challenges of working with diverse knowledge systems emerged in this project. While we endeavored to create opportunities for the expression of what laid beyond empirical observation and to not subject indigenous knowledge to evaluation by western scientific standards, we did tend to focus on environmental and ecological aspects to the exclusion of other kinds of knowledge.

Arguably, delta days did a better job in creating interactive experiences, while building bridges were more effective at creating multiple sensory experiences. Interactive experiences in delta days included large and small group discussion and artistic collaboration. These interactive opportunities were more limited in building bridges, which was more of an individual experience, although it did not preclude interaction with others. Building bridges were designed to have more multiple sensory experiences than delta days. Sound, sight, and touch were integrated into the exhibit to encourage engaging multiple senses. While art was used in delta days, it was more limited to tactile and visual experiences.

Art was intentionally designed as a boundary object (Balint and Pangaro 2017) to achieve the multiple goals of delta days and building bridges. Art was the means through which participants communicated and allowed participants to build a common understanding among themselves using shared language which evolved into new meaning through discussion, tension and reflection. It also created a safe place for unmediated expression, clarification of meaning and intention to advance the collective discourse and understanding about the deltas and the people who live there.

Both delta days and building bridges owe their transdisciplinary and transformative success to scholars and community members diving deep into work that was outside their area of comfort and expertise. Scholars with backgrounds in science participated in creating artistic expressions from cutting out nature post-it notes to being roadies during the exhibit tour. Community partners provided presentations during delta days and engaged members of their communities during the building bridges tour. Artists listened closely to scientists and community members to create accurate as well as evocative visual forms that would be meaningful to both groups. The tremendous efforts to prepare both delta days and building bridges cultivated attitudes that drew people into the hidden third; a space where different ways of knowing converged and cross-cutting sustainability goals emerged.

## Conclusions

At a time when division, rancor and polarization characterize much public debate related to sustainability among people of diverse culture and backgrounds, we need to find pathways that can allow us to communicate and understand each other. Overcoming these divides begins by recognizing and respecting different knowledges, ways of knowing and the ways we can be with each other. The failure to find respectful spaces to hold conversations, define problems and identify solutions breeds frustration, undermines trust in academic institutions and those associated with them and leads to questions about credibility, legitimacy, saliency and effectiveness of well-intentioned transdisciplinary efforts (Cash et al. 2003).

A weakness of Nicolescu’s theory (2010, 2012) of the hidden third is just that—it is a theory. It is also a theory established by a western scientist. Nevertheless, it is one that attempts to bridge “objective” and “subjective” forms of knowledge generation and hence may be useful for bridging different kinds of knowing, including indigenous and western scientific ways of knowing. This article set out to identify whether and how this space could be rendered in practice. Art is one means available that can help bridge divides among ways of knowing as it has the potential to create a level playing field for numerous types of knowledge holders. Youth, elders, land users, scientists and students, can create mutually relevant expression and understanding on more epistemologically equitable terms (Rathwell and Armitage 2016). While other scholars suggest that we need to make more room for creativity and intuition in scholarly work (Kahneman 2011; Kealiikanakaoleohaililani and Giardina 2016; Scheffer et al. 2017), there are relatively few pathways for investigating how we can practice these elements.

Art was used as a means to find an entry point into the hidden third space. Art in this project was not meant to be a conduit for transmitting science or making a political point, it was meant to create respectful space to bridge different ways of knowing and experiences while also offering the opportunity for conveying deeper spiritual and emotional connections about the importance of the deltas. Each participant expressed compassion for those living and working in other deltas (including, people, wildlife, and all beings). Art in delta days and in building bridges crossed numerous boundaries per the findings of Rathwell and Armitage (2016), including demonstrating how knowledge, practice and belief were embedded into art objects; using art to share knowledge; sharing art-making skills; providing testimony and evidence of social–ecological change in the deltas and serving as artefacts for knowledge co-production. Evidence from research suggests that art is one means of finding entry into the hidden third space, where interpretation can be open-ended and intuitive giving viewers the “power to make something different, even to be something different” (De Wolf and Lumer 2017:23).

Transdisciplinary practice seeks to work with those outside the academy. Yet, empirical examples remain scarce. Our project demonstrates how to bring together different worldviews in one collaborative space, demonstrating the opportunities for doing so and the challenges that inhibit greater connection. With such diverse cultural backgrounds, time and funding constraints and large geographic distances, opportunities for more authentic interactive experiences and unfiltered space for expression were limited. Future research that investigates how to make progress in these areas would be useful. For example, shared on-the-land experiences that include multiple sensory experiences are a promising means for achieving unfiltered spaces (e.g., Woo et al. 2007).

A key contribution of our paper is explaining five key characteristics associated with respectful transformative spaces, especially as they relate to working in collaboration with indigenous communities. That said, these five characteristics are likely applicable to other contexts when working with diverse collaborators and across cultural divides. It would useful to understand whether and how more similar cultural partnerships in transdisciplinarity would measure up against the criteria developed here.

The general idea behind trying to identify a more transformative space for transdisciplinarity is to provide opportunity for individual and collective transformative learning that is needed for sustainable change. A significant limitation of this work is that we did not collect outcome data on whether and how individuals or the collective changed. Instead, in this article we operationalized the space, where this change could take place. Further work remains to see if individual and collective change are occurring. Our collective hope is that we have made some progress in identifying where and how a transformative transdisciplinary space can be realized in the service of realizing a more social equitable and ecologically sustainable world for all.

## Footnotes

1 See: https://www.youtube.com/watch?v=R0z6loAn9kY&feature=youtu.be for a video summarizing the event. See also: http://www.usask.ca/research-groups/ddn/documents/FINAL%20Delta%20Days%20Report.pdf for a full report from the event.

2 See: https://www.youtube.com/watch?v=XHjmcdNwVpE for the video.

3 The Métis are recognized under section 35 of the Constitution Act of Canada which states, “aboriginal peoples of Canada” includes the Indian, Inuit and Métis peoples of Canada.” The advent of the fur trade in west central North America during the 18th century was accompanied by a growing number of mixed offspring of Indian women and European fur traders.  As this population established distinct communities separate from those of Indians and Europeans and married among themselves, a new Aboriginal people emerged—the Métis people—with their own unique culture, traditions, language (Michif), way of life, collective consciousness and nationhood.
